# The use of audio-biographical cues in dementia care: a four-year evaluation in Swiss hospitals, care, and domestic homes

**DOI:** 10.3389/frdem.2024.1429290

**Published:** 2024-08-30

**Authors:** Heather Edwards, Sandra Oppikofer, Damaris Aschwanden

**Affiliations:** ^1^Hellesdon Hospital, Norfolk and Suffolk NHS Foundation Trust, Norfolk, United Kingdom; ^2^Center for Gerontology, University of Zurich, Zürich, Switzerland; ^3^Center for the Interdisciplinary Study of Gerontology and Vulnerability, University of Geneva, Geneva, Switzerland

**Keywords:** dementia, biography, music, memory, emotion, relationship, person-centered care

## Abstract

**Introduction:**

In dementia care, the integration of innovative interventions is essential to enhancing the wellbeing and quality of life of people with dementia. Among these interventions, the Music Mirror intervention has emerged as a promising tool to provide personalized audio-biographical cues aimed at soothing, motivating, and engaging people with dementia. This study examined the effects of a Music Mirror intervention on the (a) wellbeing, emotions, and behavioral and psychological symptoms of 155 individuals with dementia, (b) perceived burden, relationship quality, and gains of their informal/formal caregivers, and (c) momentary closeness, wellbeing and stress of caregivers.

**Methods:**

This four-year study employed a quasi-experimental waiting-control group design, utilizing before-after measurements in Swiss hospitals, care homes, and domestic homes. For four 6-week intervention phases, Music Mirrors, i.e., brief written resources of acoustic material, associated with practical activities of daily life, were applied at least twice a week by the caregivers during critical moments such as staff handover. Repeated measures' analysis of variance and other tests were used to analyze the data.

**Results:**

Individuals with dementia had a higher wellbeing after the Music Mirror use across different care situations. While the Music Mirrors were played, individuals with dementia showed more positive than negative emotions at each measurement occasion, but emotion scores did not significantly change over time. After the MM use, caregivers felt better, closer to the person with dementia, and less stressed. Caregivers also reported significant gains at the end of the intervention. However, there were no significant changes in the frequency of the behavioral and psychological symptoms of dementia, care-related burden and relationship quality over time, regardless of the treatment condition.

**Discussion:**

By incorporating personalized audio-biographical cues into their care routines, the wellbeing of people with dementia was improved as well as it had positive momentary effects on their caregivers. The Music Mirror intervention addresses the preferences and needs of people with dementia and helps build bonds between care-recipients and caregivers. Therefore, Music Mirrors can be seen as a highly adaptive and individualized instrument to improve momentary wellbeing of people with dementia in various care situations during daily life.

## 1 Introduction

With an estimated 50 million people currently living with dementia worldwide and approximately 10 million more diagnosed each year, dementia care—in particular person-centered care—is an issue of growing importance (World Health Organization, 2021). Dementia commonly causes difficulties in navigating activities of daily life, and increasing frailty may lead to a move to residential living or hospital stays, bringing with it added stress and anxiety of changes of routine and care personnel (Aaltonen et al., 2012). The need to be understood, seen, and treated as an individual affects not just the person living with memory loss but has an impact on the quality of relationships with those involved in care and support (Nowell et al., 2013; Røsvik and Rokstad, 2020). The burden of caring may lead to exhaustion or burnout for both formal and informal carers (Costello et al., 2019). It thus is important to have strategies and/or tools that support both care-recipients and caregivers in different care environments during times of transition and uncertainty (e.g., during the move from one's own home to a care home). For example, there is an acknowledged need for information supporting personal identity to follow people through the transitions of their care as part of health and social service records (Fortinsky and Downs, 2014; Hampson and Morris, 2016). In the United Kingdom, documents of individual wishes and preferences such as *Advance Care Plans* and *This is Me* leaflets are widely used to address this issue (Petty et al., 2020). However, in countries where no such aid is available, the need remains to support the identity of people with dementia. Familiar words, sounds, or music can be powerful reminders of past experiences, both positive and negative (Jäncke, 2008). Memories and feelings associated with sound are in general retained longer than those without, even in dementia (Schaefer, 2017). If they have positive associations, they may be of practical help in supporting identity, sustaining relationships in care environments, and providing reassurance at times of transition and uncertainty (Baird and Thompson, 2018; Särkämö and Sihvonen, 2018). In terms of dementia care, the social positioning of the person with dementia is important: some researchers highlight that if the person with dementia is positively positioned and supported, the self can be maintained (Hampson and Morris, 2016). If negatively positioned, the self of the person is deconstructed to the point of being lost. Music Mirrors (MMs); i.e., brief written resources of acoustic material, associated with practical activities of daily life, are an established extension of care plans in the United Kingdom (Craig, 2020; Edwards, 2018, 2020).

MMs are positive life story memories involving sounds or music, written down briefly in someone's own words and linked to acoustic cues to reinforce their emotional significance (Edwards, 2018). MMs are based on the concept of music-evoked autobiographical memories—i.e., personal memories that are triggered by hearing music (Janata et al., 2007)—and the established evidence that music effectively evokes autobiographical memories and associated emotions in people with dementia (Baird and Samson, 2015). While music interventions have been widely adopted as a potential non-pharmacological therapy for people with dementia (Koger et al., 1999), MMs expand on such interventions due to the addition of individually important memories. Through these memories, the activated brain network is extended and may even lead to more emotional stimulation than music alone. In contrast to playlists with favorite songs, MMs are written and acoustic resources (i.e., a collection of autobiographical sequences) that also aim to facilitate the building of relationships between caregivers and care-recipients. Specifically, as resources of uniquely personal memories (e.g., the sound of rain on a caravan roof, a melody one's father whistled out of tune the bark of one's favorite dog), MMs can be used to ease the stresses of daily life, give comfort and orientation, reflect identity, and add quality to care relationships. Audio-biographical cues are collected via conversations with the person concerned and written as emails or stored as part of a care plan, with links embedded via YouTube to recorded sound. The completed MMs can be accessed on a smartphone, tablet or as information on paper without any special or personalized equipment. As such, MMs can be relatively easily integrated into daily routines of caregiving and are a low-cost intervention and can be provided when needed, not when planned (Hämäläinen et al., 2023). However, up to date, research on the MM intervention is scarce. To our best knowledge, this is the first study aiming to investigate the effects of MMs on different variables in people with dementia and their caregivers. The Center for Gerontology at the University of Zurich conducted a four-year randomized control study of the implementation of MMs in the cantons of Aargau and Basel in Switzerland. The goal of the study was to examine the effects of MMs on people with dementia and their caregivers. Specifically, we examined the effects of MMs on (a) the wellbeing, emotions, and behavioral and psychological symptoms of dementia (BPSD) of participants with dementia, (b) the perceived burden, relationship quality and gains of their caregivers, and (c) the perceived closeness between the care-recipients and informal and formal caregivers (rated by the latter) as well as the momentary wellbeing and stress of caregivers.

## 2 Materials and methods

The study was approved by the Swiss Ethics Committee on Research Involving Humans. The study was conducted according to the Swiss legal requirements, the World Medical Association Declaration of Helsinki, and the principles of Good Clinical Practice. The study was designed to gather information in real time in participants' natural environments in residential dementia care, acute hospitals, and family homes. This quasi-experimental waiting-control group study was structured in four six-week intervention phases from 2016 to 2020.

### 2.1 Procedure

The study included a baseline assessment (during 2 weeks before the start of the MM intervention), a mid-evaluation assessment (during week three and four of the MM intervention) and a post-test assessment (during the 2 weeks after the MM intervention). The MM intervention lasted 6 weeks. Over the study period, four intervention phases were conducted to respect the difficulties of participant acquisition when working with people with dementia (Sung et al., 2006). Participants were divided into a waiting-control and an intervention group for each phase according to their registration. Participants of the control group were automatically assigned to the intervention group in the next phase, so that all participants ultimately received the MMs. During the intervention phases, a MM was used by the caregivers of the intervention group at critical moments—such as staff handover, when night personnel needed to make a bond with the person with dementia, or when a patient was anxious about a change of dressing—or at minimum twice a week for at least 10 min. During the intervention phase, the control group received normal care with no MM. Measures that were assessed at baseline, mid-evaluation and post-test were completed in both the intervention and control groups (with a few exceptions, see Section 2.3).

### 2.2 Participants

For the project, three different groups of individuals were recruited, that is, (a) individuals with dementia, (b) caregivers that applied the MMs, and (c) volunteers that made the MMs.

#### 2.2.1 Individuals with dementia

People with dementia were recruited from eight research partners: care and residential homes, a specialist hospital, and an umbrella dementia organization (see Table 1). A diagnosis by a clinician was required, but etiology, duration and severity of disease were not considered as a criterion of exclusion. Potential participants were included if a screening conducted via the Mini Mental Status Examination (0–30 points) revealed 24 points or less, which is a generally accepted cutoff score indicating the presence of cognitive impairment (Folstein et al., 1975; Mitchell, 2009), with scores >19 = mild, 10–18 = moderate, <9 = severe. Exclusion criteria were schizophrenic symptoms or the need for a hearing aid. Participants were assigned to the intervention group and control group at random, but those in the control group also received the intervention eventually. Altogether, 199 individuals with dementia were recruited. Thereof, 54 individuals dropped out before or during their participation in the intervention because of moving to a different care setting, development of insuperable hearing problems or death. Participants recruited were living in nursing homes or at home alone or with their partner. The sociodemographic survey of the baseline assessment was completed for *N* = 155 individuals with dementia. However, numbers completing each measure varied across measures and measurement occasions; we report the sample sizes in brackets in the results section. The mean age of the participants was 82.54 years (SD = 9.97, Range = 55–104), and 38.5% had an Alzheimer's disease (AD) diagnosis. The sample characteristics of the persons with dementia are shown in Table 2.

**Table 1:** Recruitment.

**Care setting**	**Ambulant care**		**Long-term care**		**Acute hospital**	
	**CG**	**IG**	**CG**	**IG**	**CG**	**IG**
Intervention Phase 1 (*n* = 20)	4	6	5	5	0	0
Intervention Phase 2 (*n* = 45)	10	10	9	10	0	6
Intervention Phase 3 (*n* = 50)	0	5	23	22	0	0
Intervention Phase 4 (*n* = 84)	0	3	0	80	0	1
Sum (*N* =199)	14	24	37	117	0	7

CG, Control Group; IG, Intervention Group. A total of *N* = 199 individuals with dementia were recruited for the study. Thereof, *n* = 54 dropped out before or during the intervention phases 1–4. For a total of *N* = 155 individuals with dementia, the sociodemographic part of the baseline assessment was completed.

**Table 2:** Descriptive statistics of people with dementia at baseline.

**Variables**	**People with dementia**
Age (M, SD, range)	82.54 (9.97), 55–104
Female	70.30%
College degree or higher	17.50%
German (mother tongue)^*^	76.70%
Alzheimer's disease	38.50%
Vascular dementia	9.70%
Mixed dementia	19.40%
Other diagnosis	7.10%
Type of dementia unknown	25.20%
Antidepressants	34.80%
Neuroleptics	32.90%
Pain killers	24.50%

N = 155. ^*^Of the participants with dementia, 1.9% had French, 0.6% had Italian, and 0.6% had English as their mother tongue. Further, 7.3% reported “other” as their mother tongue and 12.9% did not report their mother tongue at all.

#### 2.2.2 Informal and formal caregivers

Ninety-nine caregivers were recruited (87.90% female, 24.20% college degree or higher). Caregivers were family members or friends (if people with dementia lived at home) and professional care staff (if people with dementia lived in nursing homes or were in the hospital). Inclusion criteria were being able to use the MM at least twice per week with at least one person with dementia and to fill in questionnaires. No sociodemographic information was collected on caregivers.

#### 2.2.3 Volunteers

Twenty-three volunteers were recruited. Inclusion criteria were: They should be interested in music, in people with dementia and be motivated to have a conversation with them. Furthermore, they should be empathic, open minded, engaged, patient, flexible to visit the people in the canton of Aargau or Basel, understand Swiss German, and have knowledge in word processing. Finally, they should also have internet access and an e-mail address. No sociodemographic information was collected on volunteers.

##### 2.2.3.1 Making of music mirrors

Volunteers attended two workshops (one on MMs and one on how to communicate with people with dementia). Then, they conducted interviews with the individuals with dementia and if necessary, with their relatives or carers, to collect biographical information. The interviews lasted a maximum of 60 min. Volunteers were instructed to take notes during the interview and importantly, to write down the exact words or quotations when important positive memories were mentioned. Based on this information, the volunteers created the MMs for the people with dementia together with the research team. The MMs consisted of four to five quotations about positive memories and their corresponding acoustic cues (e.g., spending lots of time outdoors in nature during childhood and the sound of a stream). Examples and vignettes of MMs are shown in Supplementary material. The acoustic cues serve as a connection point for memories and emotions, were downloaded from iTunes, and stored on the iPads. Volunteers revisited the people with dementia and played the MMs to confirm the content. If necessary, the MMs were adjusted. The iPads with the final MMs were handed over to the caregivers to use during the 6 weeks of intervention.

##### 2.2.3.2 Application of music mirrors

For the intervention phase, caregivers received a laminated manual with instructions on how to use the MMs. Caregivers were instructed to use the MMs at critical moments—such as staff handover, when night personnel needed to make a bond with the person with dementia, or when a patient was anxious about a change of dressing—or at minimum twice a week for at least 10 min if no critical moments occurred. Caregivers were provided with the study iPads that contained the MMs as well as an instruction video and further videos with different examples of how the MMs can be used. As one MM contains four to five memories, caregivers could choose any memory depending on the situation. For example, sometimes the memory fits the situation very well, such as when a person with dementia has a memory of hiking with their father combined with a hiking song, then the MM could be used to motivate the person for a walk. The goal of using the MM was to evoke positive emotions, distract from stress and deepen the relationship with the caregiver.

### 2.3 Measures

The outcome measures are summarized in Table 3 and briefly described below. At baseline, caregivers filled out a sociodemographic survey for people with dementia to obtain information on age, gender, mother tongue, education, musicality. living status, care setting, and medication. In general, measures that were assessed at baseline, mid-evaluation and post-test were completed in both the intervention and control groups (except the measurement of emotions and MM-related gains, see corresponding sections below). Measures that were assessed before and after each use of the MMs were completed in the intervention group only. Internal consistency (Cronbach alpha) is reported for measures that were assessed at baseline. Table 4 provides an overview of the descriptive statistics of the outcome measures at baseline and post-test depending on group membership.

**Table 3:** Outcome measures.

**Outcome**	**Measurement**	**Reference**	**Rated by**	**Rated when**
BPSD	Neuropsychiatric inventory (NPI)	Reuther et al., 2016	Caregivers (IG, CG)	Baseline, mid-evaluation, post-test
Emotions	Observed emotion rating scale (OERS)	Lawton et al., 1999	Student assistants, researchers (IG)	Five-minute observations while applying the music mirrors at baseline, mid-evaluation, post-test
Wellbeing	Diary	Visual Scale, Supplementary Figure 1	People with dementia or caregivers (IG)	Before and after each use of music mirrors
Caregiver-related burden and gains	Caregiver distress scale (CDS)	Cousins et al., 2002	Caregivers (IG, CG)	Baseline, mid-evaluation, post-test
	Gain in Alzheimer care instrument (GAIN)	Yap et al., 2010	Caregivers (IG)	Post-test
Acute stress	Diary	Single item	Caregivers (IG)	Before and after each use of music mirrors
Closeness	Diary	Single item	Caregivers (IG)	Before and after each use of music mirrors
Relationship quality	Six self-generated items	Authors	Caregivers (IG, CG)	Baseline, mid-evaluation, post-test

BPSD, Behavioral and psychological symptoms of dementia. IG, intervention group, CG, control group. IG and CG refers to in which group the measure was conducted.

**Table 4:** Descriptive statistics of measures.

**Variables**	**Baseline IG**	**Baseline CG**	**Mid-evaluation IG**	**Mid-evaluation CG**	**Post-test IG**	**Post-test CG**	**Range**
Aggression (BPSD, measured with the NPI)	1.59 (1.55)	2.10 (2.15)	1.48 (1.40)	1.52 (1.66)	1.39 (1.42)	1.71 (1.56)	1–4
Depressive mood (BPSD, measured with the NPI)	1.54 (1.59)	1.76 (1.75)	1.25 (1.33)	1.44 (1.69)	1.08 (1.26)	1.82 (1.56)	1–4
Apathy (BPSD, measured with the NPI)	1.48 (1.62)	1.45 (1.68)	1.20 (1.45)	1.20 (1.58)	1.30 (1.53)	1.32 (1.52)	1–4
Irritability (BPSD, measured with the NPI)	1.68 (1.57)	2.09 (1.49)	1.40 (1.54)	1.64 (1.66)	1.37 (1.69)	1.86 (1.56)	1–4
Aberrant motor behavior (BPSD, measured with the NPI)	1.35 (1.66)	2.21 (1.80)	1.39 (1.74)	1.68 (1.84)	1.17 (1.59)	1.93 (1.74)	1–4
Emotions (measured with the OERS)	11.86 (10.49)	NA	8.09 (6.70)	NA	7.83 (6.66)	NA	−24–24
Caregiver burden (measured with the CDS)	1.15 (0.88)	1.74 (0.87)	1.24 (0.78)	1.92 (0.85)	1.04 (0.86)	1.74 (0.89)	0–4
MM-related gains (measured with the adapted GAIN)	NA	NA	NA	NA	2.57	NA	0 to 4
Relationship quality (measured with self-generated items)	8.16 (1.42)	7.84 (1.55)	7.85 (1.72)	7.26 (2.23)	8.08 (1.37)	8.32 (1.38)	1–10
**Diary**	**Before MM**	**After MM**	**Range**				
Wellbeing of people with dementia (visual scale)	3.00 (0.72)	2.15 (0.55)	1 to 6				
Wellbeing of caregivers (visual scale)	1.77 (0.77)	1.46 (0.58)	1 to 6				
Closeness (one item)	2.41 (0.89)	2.88 (0.87)	1 to 5				
Stress (one item)	1.30 (0.66)	1.09 (0.48)	1 to 6				

The numbers reported in the table refer to the mean with standard deviations in brackets. BPSD, behavioral and psychological symptoms of dementia (frequency); NPI, neuropsychiatric inventory; OERS, observed emotion rating scale; CDS, caregiver distress scale; MM, music mirrors; GAIN, gain in Alzheimer care inventory; IG, intervention group; CG, control group; NA, not applicable as the variable was not assessed; MM, Music Mirror. Note that for wellbeing, lower scores reflect better wellbeing.

#### 2.3.1 Behavioral and psychological symptoms of dementia (BPSD)

At baseline, mid-evaluation and post-test, caregivers in both the intervention and control groups were asked whether there are any phases during which the person with dementia refuses to cooperate or to be taken care of. If so, caregivers subsequently rated five symptoms (i.e., restless, apathetic, irritated, depressed mood, aggressive) from the neuropsychiatric inventory (NPI) (Reuther et al., 2016). Caregivers rated if the symptom was present (yes/no) and if yes, how frequent it occurred (on a scale from 1 = seldom to 4 = very often), how severe it was (on a scale from 1 = mild to 3 = severe) and how stressful it was for the caregiver (on a scale from 0 = not at all to 5 = extreme). Cronbach's alphas ranged from 0.60 (severity) to 0.71 (stress) at baseline, indicating acceptable internal consistency.

#### 2.3.2 Emotions

At baseline, mid-evaluation and post-test, student assistants and researchers applied the Observed Emotion Rating Scale (OERS; Lawton et al., 1999) while caregivers played the MMs for the people with dementia in the intervention group. The OERS was used to observe five emotions in people with dementia, three negative (anger, anxiety, sadness) and two positive (pleasure, interest), which are derived from Ekman's universal basic emotions theory (Ekman and Friesen, 1971). The appearance of these emotions was rated for approximately five minutes on a five-point Likert scale, ranging from “never” to “more than three minutes”. To calculate a sum score, the points of positive and negative emotions were added separately, then weighted, and finally, the negative emotion score was deducted from the positive emotion score. A positive sum score indicates that the person with dementia exhibits more positive emotions relative to negative emotions. Cronbach's alpha was 0.73 at baseline, indicating acceptable internal consistency.

#### 2.3.3 Wellbeing

Before and after each use of the MMs, the well-being of people with dementia in the intervention group was assessed using an alteration of the Dementia Mood Picture Test (Tappen and Barry, 1995) by the person with dementia him-or herself or the caregiver. People with dementia were shown six different pictures in six simple line drawings of a face (see Supplementary Figure 1) by the caregivers. The faces depicted expressions on a six-point Likert scale, ranging from happy (1) to sad (6). Lower scores reflect higher well-being. People with dementia were asked to point on the face that currently reflects their mood best. The caregiver noted the answer accordingly. If people with dementia could not rate the pictures themselves due to severe cognitive impairment, the caregivers rated the current well-being of people with dementia.

#### 2.3.4 Caregiver burden and gains

At baseline, mid-evaluation and post-test, the Caregiver Distress Scale (CDS; Cousins et al., 2002) was used to assess potential caregiver burden in both the intervention and control groups. The scale contains 17 items that were rated on a five-point Likert scale ranging from 0 (strongly disagree) to 4 (strongly agree). Cronbach's alpha was 0.94 at baseline, indicating high internal consistency.

Likewise, an adapted version of the Gain in Alzheimer Care Inventory (GAIN; Yap et al., 2010) was used to measure MM-related gains at post-test in the intervention group only. The GAIN was adapted such that the items referred to the MM intervention. Specifically, each item started with “The use of the music mirror in people with dementia…” and was continued by the original GAIN items (e.g., “… increased my patience and made me to a more understanding person”). Caregivers reported on all 10 adjusted GAIN items on a five-point Likert scale ranging from 0 (strongly disagree) to 4 (strongly agree). Cronbach's alpha was 0.94 at baseline, indicating high internal consistency.

#### 2.3.5 Relationship quality

At baseline, mid-evaluation and post-test, caregivers reported on their relationship satisfaction with the person with dementia in both the intervention and control groups. The six items were designed by the researchers (e.g., “I am satisfied with the contact to the care-recipient”) and answered on a scale from 1 (total agreement) to 10 (total rejection). The items are listed in Supplemental material. Cronbach's alpha was 0.89 at baseline, indicating high internal consistency.

#### 2.3.6 Diary

During the intervention phase, a diary consisting of a short questionnaire was filled out by the caregivers each time after they had used the MM. Over the four intervention phases, 1,406 diary entries were collected. The goal of the diary was to collect information on various variables in real time in the participants' natural environment (Mehl et al., 2014). In the diary, caregivers reported first on the state (i.e., depressive, apathetic, aggressive, irritated, restless) in which the person with dementia was immediately before the MM application. Second, caregivers reported in which situation the MM was applied (i.e., medication, meals, doctor's appointment, transfer, nursing care, change of caregivers, evening rest, and something else). Third, caregivers rated (or helped to rate) the wellbeing of the person with dementia and for themselves before and after the MM application on the visual analog scale described above Tappen and Barry (1995). Fourth, caregivers rated their stress levels (one item) before and after the MM application on a scale from 1 (“not stressed at all”) to 6 (“very highly stressed”). Fifth, caregivers reported which emotions (i.e., anger, fear, sadness, joy, alertness) were evoked how strongly (1 = not at all, 5 = very strongly) through the MM application in the person with dementia. Finally, caregivers rated their perceived closeness (one item) with the person with dementia before and after the MM application on a scale from 1 (“not close at all”) to 5 (“very close”). In addition, caregivers had the opportunity to write down any comments (e.g., reason for early demolition, observations made during the application).

### 2.4 Statistical analyses

#### 2.4.1 Power analysis

A power analysis using G^*^Power (Faul et al., 2007) was conducted to determine the sample size. The effect sizes used for the power analysis are based on a meta-analysis of music therapy for dementia with dependent variables such as agitation/relaxation, cooperation, positive/negative affect, social interaction and cognitive/dementia-related measures (Koger et al., 1999). The mean effect size of Koger et al.'s meta-analysis was *d* = 0.788, corresponding to a f-value of 0.349. Depending on the statistical analysis (e.g., generalized linear model, one-sample *t*-test), the sample should comprise at least 15 (one-sample *t*-test) to 31 (generalized linear model) individuals. The power analysis indicated that a total sample size of 31 participants would be necessary to detect a moderate-to-large effect size with 80% power at the 5% significance level.

#### 2.4.2 Tests used for analyses

Normal distribution was tested, and parametric methods of analysis were used if applicable. For ordinally scaled items, scale and subscale median scores were used in place of missing item values. A repeated-measures' *t*-test was used to examine whether there is a significant difference in the momentary wellbeing (diary data) of individuals with dementia before and after the use of MMs. Using a generalized linear model, we explored whether the effect of the MM intervention on momentary wellbeing varied depending on seven care situations (medication, meal, doctor's visit, nursing care action, change of caregivers, evening rest, another situation). One-sample *t*-tests were used to examine whether people with dementia showed more positive relative to negative emotions while listening to the MMs at baseline, mid-evaluation, and post-test. Using the *t*-tests, we tested whether the mean differences in the emotion scores (OERS) at different time points (baseline, mid-evaluation, post-test) were significantly different from zero. If the mean differences differ positively (vs. negatively) from zero, people with dementia show more positive (vs. negative) emotions while listening to the MMs. In addition, we used a one-way repeated measures' analysis of variance (ANOVA) to test whether the emotion scores significantly changed over time in the intervention group. A two-way repeated measures ANOVA was used to examine the effects of treatment (intervention, control) and time (baseline, mid-evaluation, post-test) on each of the BPSD (NPI). For the data of caregivers, repeated-measures' *t*-tests were used to examine whether there were significant differences in the momentary wellbeing, closeness and stress (all diary data) of caregivers before and after the use of MMs. Two-way repeated measures ANOVAs were run to examine the effects of treatment (intervention, control) and time (baseline, mid-evaluation, post-test) on care-related burden and relationship quality. One-sample *t*-tests were used to examine whether caregivers in the intervention group reported any gains (GAIN) from the use of MMs at post-test. All analyses controlled for education. We did not control for etiology of observed cognitive impairment nor gender as there is no evidence suggesting a meaningful influence on results. Unstandardized coefficients and *p*-values are reported.

## 3 Results

### 3.1 Effects of the MM intervention on people with dementia

The repeated-measures *t*-test showed a significant positive effect of the MM application on the momentary wellbeing (measured using the visual scale in the diary) of people with dementia. After the MM use, people with dementia (*n* = 125) reported a 0.9-point better wellbeing (measured on the 6-point visual scale in the diary) than before the MM use (*t* = 7.69, *p* < 0.001; before: *M* = 3.00, *SD* = 0.72 vs. after: *M* = 2.15, *SD* = 0.55; lower scores reflect better wellbeing). The effect size was *d* = 0.65, referring to a moderate effect (Cohen, 1992). Our exploration analysis (using a generalized linear model) showed that the effect of the MM intervention remained significant across different care situations (medication, meal, doctor's visit, nursing care action, change of caregiver, evening rest, another situation; *n* = 125, B = 1.07, SE = 0.6, t = 19.39, *p* < 0.001). This means, the wellbeing of people with dementia was higher after (vs. before) the MM use irrespective of different care situations.

Moreover, the mean differences in the emotion scores (measured using the OERS) were significantly different from zero at baseline (*n* = 31, *t* = 3.99, mean difference: 0.72, *p* < 0.001), mid-evaluation (*n* = 29, *t* = 4.56, mean difference: 0.53, *p* < 0.001), and post-test (*n* = 28, *t* = 5.10, mean difference: 0.59, *p* < 0.001). This means, student assistants and researchers observed more positive (vs. negative) emotions in the individuals with dementia while the caregivers played their MMs for them. The analysis was repeated with a subgroup of people with severe cognitive impairment (MMSE <5; *n* = 20 at baseline, *n* = 19 at mid-evaluation, *n* = 18 at post-test), and results remained the same (all *p* < 0.004). This means, the MM contributed to positive emotions regardless of the severity of cognitive impairment. However, the emotion scores did not significantly change over the three measurement occasions (one-way repeated measures ANOVA: *n* = 28, *F*_(1.37, 27.45)_ = 0.031, *p* = 0.922, η^2^ = 0.002). Likewise, there were no significant changes in the frequency of any of the five BPSD (measured using the NPI) over time, regardless of the treatment condition. Additionally, there was no interaction effect, indicating that the MM intervention did not differentially affect changes in the BPSD over time. Specifically, for aggression, the two-way repeated measures ANOVA revealed no significant main effects of neither treatment [F_(1, 12)_ = 1.240, *p* = 0.287, η^2^ = 0.094] nor time [F_(2, 24)_ = 1.044, *p* = 0.368, η^2^ = 0.080], and no significant time x treatment interaction [F_(2, 24)_ = 0.915, *p* = 0.414, η^2^ = 0.071]. For depressive mood, the two-way repeated measures ANOVA showed no significant main effects of neither treatment [F_(1, 6)_ = 0.140, *p* = 0.721, η^2^ = 0.023] nor time [F_(2, 12)_ = 0.329, *p* = 0.726, η^2^ = 0.052], and no significant time x treatment interaction [F_(2, 12)_ = 2.183, *p* = 0.155, η^2^ = 0.267]. For apathy, the two-way repeated measures ANOVA showed no significant main effects of neither treatment [F_(1, 7)_ = 0.488, *p* = 0.507, η^2^ = 0.065] nor time [F_(2, 14)_ = 0.116, *p* = 0.891, η^2^ = 0.016], and no significant time x treatment interaction [F_(2, 14)_ = 0.685, *p* = 0.520, η^2^ = 0.089]. For irritability, the two-way repeated measures ANOVA revealed no significant main effects of neither treatment [F_(1, 7)_ = 0.733, *p* = 0.420, η^2^ = 0.095] nor time [F_(2, 14)_ = 0.167, *p* = 0.848, η^2^ = 0.023], and no significant time x treatment interaction [F_(2, 14)_ = 0.607, *p* = 0.559, η^2^ = 0.080]. Finally, for aberrant motor behavior (such as restlessness, repeatedly opening drawers, and pulling at clothing), the two-way repeated measures ANOVA showed no significant main effects of neither treatment [F_(1, 7)_ = 0.799, *p* = 0.401, η^2^ = 0.102] nor time [F_(2, 14)_ = 0.773, *p* = 0.480, η^2^ = 0.099], and no significant time x treatment interaction [F_(2, 14)_ = 0.407, *p* = 0.673, η^2^ = 0.055].

### 3.2 Effects of the MM intervention on caregivers

There were no significant changes in care-related burden (assessed using the CDS) and relationship quality (assessed using the six self-generated items) over time, regardless of the treatment condition. There were neither any interaction effects, indicating that the MM intervention did not differentially affect changes in care-related burden and relationship quality over time. For care-related burden, the two-way repeated measures ANOVA revealed no significant main effects of neither treatment [F_(1, 102)_ = 1.562, *p* = 0.214, η^2^ = 0.015] nor time [F_(2, 204)_ = 0.695, *p* = 0.500, η^2^ = 0.007], and no significant time x treatment interaction [F_(2, 204)_ = 2.133, *p* = 0.121, η^2^ = 0.020]. For relationship quality, the two-way repeated measures ANOVA showed no significant main effects of neither treatment [F_(1, 102)_ = 3.044, *p* = 0.084, η^2^ = 0.029] nor time [F_(2, 204)_ = 0.045, *p* = 0.888, η^2^ = 0.000], and no significant time x treatment interaction [F_(2, 204)_ = 1.205, *p* = 0.288, η^2^ = 0.012]. Of note, the relationship quality was already relatively high at baseline in both groups (see Table 4). Nevertheless, caregivers reported MM-related gains at post-test (assessed using the adapted GAIN; *n* = 67, t = 19.96, mean difference: 2.29, *p* < 0.001). Moreover, the use of MMs had positive momentary effects on the caregivers: caregivers (*N* = 99) felt closer to the person with dementia (almost +0.5-point on a 5-point-scale, assessed using one item in the diary) after the MM use (*t* = −4.26, *p* < 0.001; before: *M* = 2.41, *SD* = 0.89 vs. after: *M* = 2.88, *SD* = 0.87). The effect size was moderate (*d* = 0.49). Caregivers also reported a 0.3-point better wellbeing (measured on the 6-point visual scale in the diary) after the MM use (*t* = 6.58, *p* < 0.001; before: *M* = 1.77, *SD* = 0.77 vs. after: *M* = 1.46, *SD* = 0.58; lower scores reflect better wellbeing). The effect size was *d* = 0.46, referring to a moderate effect. Likewise, caregiver felt less stressed (-0.2-point on a 6-point-scale, assessed using one item in the diary) after the MM use (*t* = 6.41, *p* < 0.001; before: *M* = 1.30, *SD* = 0.66 vs. after: *M* = 1.09, *SD* = 0.48). The effect size was small to moderate (*d* = 0.33).

Finally, researchers reached out to caregivers after the study again for a short follow-up survey. Of 29% of caregivers who participated, 62% have used the MM at least once a month after study completion. Although 64% said that they lacked the technical resources to use the MM fully, only 6% of those questioned would not recommend MMs to others. In two cases, MMs had aroused negative emotions in the individuals with dementia, or the person with dementia had lost interest.

## 4 Discussion

The goal of the study was to examine the effects of MM on the (a) wellbeing, emotions, and behavioral and psychological symptoms of dementia (BPSD) of participants with dementia, (b) perceived burden, relationship quality and gains of their caregivers, and (c) momentary closeness, wellbeing and stress of caregivers. The findings showed that, on average, people with dementia had a better wellbeing after the MM use, across different care situations. Individuals with dementia also showed more positive than negative emotions while the MMs were played at each measurement occasion. However, the emotions did not significantly change over the intervention period. Although the MMs evoked more positive than negative emotions at each measurement occasion, these effects seemed to be rather short-term (i.e., in the moment) as they did not lead to any longer-term change such as significantly more positive emotions at the end vs. at the beginning of the intervention. Likewise, there were no significant changes in the frequency of any of the five BPSD over time, regardless of the treatment condition. Additionally, there was no interaction effect, indicating that the MM intervention did not differentially affect changes in the BPSD over time. However, the use of MMs had positive momentary effects on the caregivers, such that they felt (a) better, (b) closer to the person with dementia, and (c) less stressed after the MM use. Caregivers also reported significant MM-related gains at post-test, but there were no significant changes in care-related burden and relationship quality over time, regardless of the treatment condition. As such, the effects of the MM seem to be rather short-term (i.e., in the moment) than long-term on both people with dementia and their caregivers, but with moderate effect sizes. The use of MMs can be seen as a highly adaptive and individualized way to improve momentary wellbeing in people with dementia, when different behavioral and psychological symptoms of dementia occur and in various situations of daily life.

The findings of the present study are in line with other research, showing that music can evoke biographical memory and associated emotions in people with dementia (Baird and Thompson, 2018; Ridder et al., 2023). Moreover, it has been found that brain regions which are active when musical memory is encoded correspond to areas with minimal cortical degeneration and minimal disruption of glucose-metabolism in AD patients (Jacobsen et al., 2015). Another group of researchers showed that musical evoked audio-biographic memories were not only significantly more specific than memories retrieved in silence, but also retrieved significantly faster in people with AD (El Haj et al., 2012). The present work adds to the existing literature that audio-biographical cues (i.e., MMs) in various contexts of care led to positive outcomes in both people with dementia and their caregivers. MMs offered ways to ease and defuse difficult moments of care and further granted insights into behaviors and motivations. This encouraged enhanced social interactions and better understanding between people with dementia and their caregivers. Caregivers themselves experienced temporary benefits in increased wellbeing and reduced sense of acute stress. This suggests that the use of MMs strengthens the momentary connection of the person with dementia and their caregiver. However, the MM intervention did not reduce the care-related burden of caregivers. This may be because caring situations can be inherently challenging and difficult. Nevertheless, MMs seem to promote resilience, such that caregivers reported MM-related gains, suggesting that the MM intervention has the potential to support personal growth of caregivers. Caregivers may benefit in terms of personal development, which may help them to deal with acute stress situations. Of note, the majority of participants intended to use MMs beyond the duration of the study. The MMs concept of personal resources of audio-biographical cues was found to be a valuable practical tool in enhancing the quality of relationships in dementia care, and relevant and transferable to Swiss care contexts.

Limitations of the study are the difficulty of recruiting people living at home as well as in hospitals (cf. Table 1). There are several reasons why recruitment and the implementation of MMs may be more challenging in these settings compared to acute and long-term care: Domestic caregivers that live together with the person with dementia may be under significant stress and may not feel open to trying new or unfamiliar interventions. Likewise, hospitals are often fast paced with a focus on immediate medical treatment. The urgent nature of care can make it difficult to prioritize or integrate complementary interventions like MMs. Moreover, hospitalized patients often have severe or critical health conditions, which may limit their ability to participate or engage in MMs. Hospitals may also face staffing shortages, and allocating time for MMs may be seen as less critical compared to essential medical care. In addition, limited space within hospitals may contribute to the difficulty of implementing MMs, especially if patients share rooms. Further studies could work with music therapists and other (external) healthcare professionals to integrate MMs into holistic care of hospital patients. Furthermore, we did not use individualized measurements. In future work, it is recommended to explore individual goals as outcome measures (Clare et al., 2019). An additional possibility would be to monitor target complaints: change of severity or degree of improvement as methods for scoring (Donnelly and Carswell, 2002). Likewise, physical measures for an objective just-in-time adaptation and outcome measure could be of interest. In addition, internal validity could be increased by conducting a randomized controlled trial, whereas our sampling was non-random. We aimed to evaluate the effectiveness of a specific intervention, which we believed to be effective, and ensured that this information was not withheld from individuals with dementia. The main question was therefore on how to find an appropriate balance between scientific ambition, ethics, and feasibility. Additionally, the research question required testing in a natural setting for higher external validity. We thus chose a quasi-experimental design and refrained from conducting a randomized control trial. Moreover, if participants were assigned to the control group, they were assigned to the intervention group during the next phase; however, this means that those participants had to wait several months (up to 12) until they could participate in the intervention. Future studies may adapt their study design, such that the participation in the intervention is possible directly upon completion of the control group phase, and the wait gets reduced to 6 weeks only.

In future studies, it would be interesting to investigate how individualized MMs change over time and whether there are specific situations, characteristics, and contexts in which they are particularly effective. Furthermore, it would be interesting to find out for which other groups of people MMs could help to build and stabilize relationships and wellbeing (e.g., in the disability sector).

To conclude, MMs are just-in-time adaptive interventions as they offer support at the right time (i.e., when needed) and in the right quantities (i.e., as long as requested) (Nahum-Shani et al., 2015). The use of MMs is a form of a highly individualized intervention, which has the potential to enable people to do what they have reason to value (World Health Organization, 2015). It addresses preferences and needs of people with dementia, enhances their identity and social participation and helps to build bonds between carers and care-recipients. For individuals with late-stage dementia, such non-verbal communication is crucial for person-centered care to succeed in meeting their psychological needs (Ridder et al., 2023). MMs are therefore in line toward a more person-centered and innovative approach of long-term care for people with dementia.

## Data Availability

The datasets presented in this article are not readily available because the data cannot be made publicly available for legal and ethical reasons. Unfortunately, the disclosure to third parties was not included in the declaration of consent during the data collection process. Requests to access the datasets should be directed to sandra.oppikofer@zfg.uzh.ch.
