# Supplementary material for: The use of audio-biographical cues in dementia care: a four-year evaluation in Swiss hospitals, care, and domestic homes
Source: Front Dement. 2024 Aug 30;3:1429290. doi: 10.3389/frdem.2024.1429290 (PMC11401044; doi:10.3389/frdem.2024.1429290)
Supplement: Supplementary file 1 [file Table_1.DOCX]

Supplementary Material

# Autobiographic and sound examples of Music Mirrors

1. A man talked about how he met his wife: "I met my wife on the football pitch. I was a goalie for FC Wettingen."

Remembered song: 'Er steht im Tor' by Wencke Myhr <https://www.zfg.uzh.ch/static/musikspiegel/lied.html>

1. A woman talked about her childhood: "I spent a lot of time outdoors in nature as a child. I have very fond memories of the Engelberger Aar, it always helped me to fall asleep."

   Remembered sound: ‘the sound of a stream – the Engelberger Aar’

<https://www.zfg.uzh.ch/static/musikspiegel/bach.html>

**2 Vignettes**

1. Mrs. A. is reluctant to socialize with other care home residents, and often asks to withdraw to sit alone in her room. A carer, using the MM, plays the sound of church bells, and she immediately recognizes the carillon as the one from her village. "That's where I came from," she says happily, and begins to talk with animation. Later, feeling reassured and more confident, she agrees to rejoin the social group in the lounge.
2. Mrs. D., an elderly resident in dementia care often forgets to eat and drink and must be prompted by staff to take food and liquids. A carer reminds her that she used to love dancing with her husband and, using the MM, plays a favorite, slightly silly song. They begin to dance together, at first slightly stiffly and then with real warmth. Afterwards the carer suggests that “after all that exercise” they must both be thirsty, and the resident accepts a drink with enthusiasm. Here the MM is used to change a situation of potential confrontation to one where drinking with a friend comes naturally after a dance.
3. Mr. M. is an in-patient in a hospital acute geriatric ward. A urinary infection makes him feel unwell and sleepy and he finds it hard to move or open his eyes when a nurse asks him to accompany her to the treatment room. Using the MM, the nurse reminds him of a song he sang with his father on hiking trips. At the mention of his father, he opens his eyes, nods in agreement and finally, with the shadow of a smile, makes the great effort to stand up and walk with her.

**3 Wellbeing**

The wellbeing of people with dementia was assessed using an alteration of the Dementia Mood Picture Test (Tappen and Barry 1995). People with dementia were shown six different pictures in six simple line drawings of a face (see supplemental material). The faces depicted expressions on a six-point Likert scale, ranging from happy to sad. The pictures were shown to the people with dementia, and they were asked to point on the face that currently reflects their mood best.


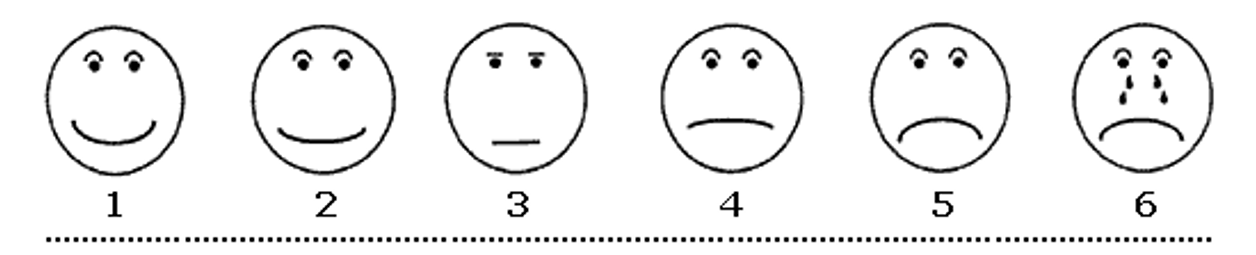


*Figure S1*. Faces depicting expressions on a six-point Likert scale, ranging from happy (1) to sad (6).

**4 Six self-generated items on relationship satisfaction**

At baseline, mid-evaluation, and post-test, caregivers reported on their relationship satisfaction with the person with dementia (“care-recipient”) using six items on a Likert scale from 1 (total agreement) to 10 (total rejection). The items were designed by the researchers and are shown below. The internal consistency was very high (Cronbach Alpha α = 0.919).

**4.1 Self-generated items**
“Please tell us about your relationship with the person you care for (“care-recipient”). Please check the appropriate box, where 1 stands for total disagreement and 10 for total agreement.”

1. I am satisfied with the contact to the care-recipient.
2. I understand the care-recipient well.
3. I feel close to the care-recipient.
4. I feel good when I am in contact with the care-recipient.
5. I know the care-recipient well.
6. I understand the way the care-recipient behaves.
